# Assessment of Motor Unit Potentials Duration as the Biomarker of DT-DEC01 Cell Therapy Efficacy in Duchenne Muscular Dystrophy Patients up to 12 Months After Systemic–Intraosseous Administration

**DOI:** 10.1007/s00005-023-00691-y

**Published:** 2023-11-24

**Authors:** Adam Niezgoda, Grzegorz Biegański, Jacek Wachowiak, Jarosław Czarnota, Krzysztof Siemionow, Ahlke Heydemann, Anna Ziemiecka, Maria H. Sikorska, Katarzyna Bożyk, Maria Siemionow

**Affiliations:** 1 Department of Neurology, Poznan University of Medical Sciences, Poznan, Poland; 2 Department of Infectious Diseases and Child Neurology, Poznan University of Medical Sciences, Poznan, Poland; 3 Department of Pediatric Oncology, Hematology and Transplantology, Poznan University of Medical Sciences, Poznan, Poland; 4 Hospital MedPolonia, Poznan, Poland; 5 Dystrogen Therapeutics Corp., Chicago, IL USA; 6 Department of Orthopaedics, University of Illinois at Chicago, Chicago, IL USA; 7 Department of Physiology and Biophysics, University of Illinois at Chicago, Chicago, IL USA; 8 Center for Cardiovascular Research, University of Illinois at Chicago, Chicago, IL USA; 9 Chair and Department of Traumatology, Orthopedics and Surgery of the Hand, Poznan University of Medical Sciences, Poznan, Poland

**Keywords:** Stem cell therapy, Duchenne muscular dystrophy, Dystrophin Expressing Chimeric (DEC) cell, Safety, Electromyography (EMG), Biomarker

## Abstract

Duchenne muscular dystrophy (DMD) is a lethal X-linked disease caused by mutations in the dystrophin gene, leading to muscle degeneration and wasting. Electromyography (EMG) is an objective electrophysiological biomarker of muscle fiber function in muscular dystrophies. A novel, DT-DEC01 therapy, consisting of Dystrophin Expressing Chimeric (DEC) cells created by fusing allogeneic myoblasts from normal donors with autologous myoblasts from DMD-affected patients, was assessed for safety and preliminary efficacy in boys of age 6–15 years old (*n* = 3). Assessments included EMG testing of selected muscles of upper (deltoideus, biceps brachii) and lower (rectus femoris and gastrocnemius) extremities at the screening visit and at 3, 6, and 12 months following systemic–intraosseous administration of a single low dose of DT-DEC01 therapy (Bioethics Committee approval no. 46/2019). No immunosuppression was administered. Safety of DT-DEC01 was confirmed by the lack of therapy-related Adverse Events or Serious Adverse Events up to 22 months following DT-DEC01 administration. EMG of selected muscles of both, ambulatory and non-ambulatory patients confirmed preliminary efficacy of DT-DEC01 therapy by an increase in motor unit potentials (MUP) duration, amplitudes, and polyphasic MUPs at 12 months. This study confirmed EMG as a reliable and objective biomarker of functional assessment in DMD patients after intraosseous administration of the novel DT-DEC01 therapy.

## Introduction

Duchenne muscular dystrophy (DMD) is a lethal X-linked disease caused by mutations in the dystrophin gene, resulting in muscle degeneration, wasting, and weakness. Currently, no therapy exists to cure or halt the progression of the disease, making the development and testing of new therapeutic approaches essential. We have developed a novel DT-DEC01 therapy, which is based on Dystrophin Expressing Chimeric (DEC) cells created by fusing human myoblasts derived from normal (allogeneic) and DMD-affected (autologous) donors. We have previously reported the long-term efficacy and safety of DEC therapy in preclinical *mdx* mice models of DMD (Heydemann and Siemionow 2023; Siemionow et al. 2018a, b, 2019; 2022a, b). Moreover, recently, we have reported, both the safety and the preliminary efficacy of DT-DEC01 therapy in the first-in-human pilot study assessed in DMD patients, at 6 and 12 months after systemic intraosseous administration (Heydemann et al. 2023; Siemionow et al. 2023).

To confirm the long-term efficacy of new therapeutic approaches, it is important to choose appropriate tools for functional assessments. However, objective confirmation of therapy efficacy in DMD patients poses a major challenge. The most commonly used functional tests, such as the 6-Minute Walk Test (6MWT), North Star Ambulatory Assessment (NSAA), or Performance of Upper Limb (PUL 2.0), are dependent on the patient’s health status, mood, feelings, and willingness to perform the tasks, and therefore are not fully objective, despite being well-established in DMD clinical studies (Goemans et al. 2013; Mazzone et al. 2010, 2013; McDonald et al. 2010, 2013, 2022).

To address this limitation, we searched for functional assessments that are independent of the patient’s influence. An example of an objective and well-accepted methods used for evaluation of DMD patients is electromyography (EMG) (Derry et al. 2012; Klimczak et al. 2020; Szmidt-Sałkowska et al. 2015; Verma et al. 2017).

Electromyography is an objective electrophysiological biomarker of muscle fiber function and has been used extensively to study muscular dystrophies (Derry et al. 2012; Klimczak et al. 2020; Ropars et al. 2016; Szmidt-Sałkowska et al. 2015; Verma et al. 2017). While there are reports on the functional assessment of the lower (Frigo and Crenna 2009; Ropars et al. 2016; Vandekerckhove et al. 2020) and upper extremities (Janssen et al. 2020; Trost et al. 2021) of DMD patients using surface EMG, a method commonly used in kinesiology (Janssen et al. 2020; Lobo-Prat et al. 2017; Nizamis et al. 2020), there are several limitations to this approach, such as the lack of quantitative assessment of changes in the single motor unit potential (MUP). To overcome this limitation, we applied needle EMG as a well-established, minimally invasive, and patient-independent method of skeletal muscle evaluation, to assess the efficacy of the DT-DEC01 therapy (Derry et al. 2012; Heydemann et al. 2023; Katirji 2007; Klimczak et al. 2020; Szmidt-Sałkowska et al. 2015; Verma et al. 2017).

For the confirmation of therapy efficacy at the molecular level, assessment of dystrophin expression by Western Blot in the muscle samples taken from patients’ biopsies is commonly considered as an assessment required by the regulatory bodies (Aartsma-Rus et al. 2019). However, open muscle biopsy is an invasive procedure performed under anesthesia that carries potential risks for DMD patients (van den Bersselaar et al. 2022). Therefore, in a search for less invasive and safer methods of assessment of efficacy of novel therapeutic approaches, we propose the use of EMG as a quantitative and objective biomarker, assessing electrophysiological changes occurring in DMD-affected muscles before and after DT-DEC01 therapy administration.

In our pilot clinical study, we confirmed the role of EMG assessment by evaluating standard EMG parameters of MUP duration and amplitudes over a 6-month follow-up, revealing progressive improvements in these electrophysiological biomarkers in the selected muscles of both ambulatory and non-ambulatory patients (Heydemann et al. 2023). To further confirm the long-term value of EMG as the biomarker of electrophysiological changes occurring in DMD, in the current study, we have confirmed the efficacy of DT-DEC01 therapy by standard needle EMG assessment up to 12 months after systemic–intraosseous administration of a single, low dose of DT-DEC01 to DMD patients.

## Materials and Methods

### Study Design

This study was a single-site pilot study that assessed safety and efficacy of a systemic–intraosseous administration of a single dose of DT-DEC01 in male patients of age 5–18 years old with genetically confirmed DMD. The study protocol calls for ten patients to be enrolled. Study protocol (DT-DEC01-DMD) was compliant with the Good Clinical Practice and was approved by the Bioethics Committee at the Regional Medical Council in Poznan, Poland (approval no. 46/2019). The study was conducted in accordance with the Declaration of Helsinki. All patients, their parents, and the donors provided informed consent for participation in the study.

**Fig. 1:**
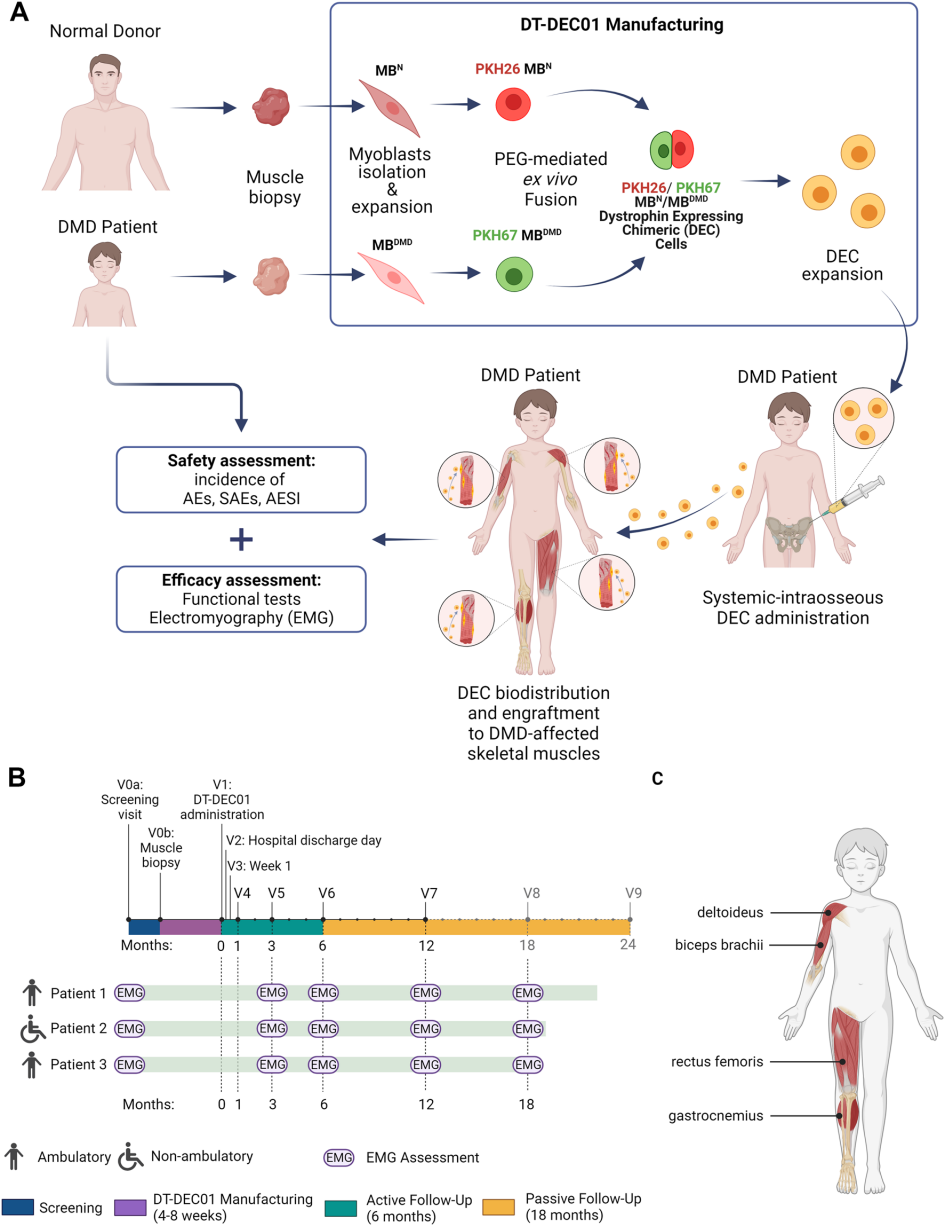
The outline of the first-in-human pilot study assessing safety and efficacy of the systemic–intraosseous administration of DT-DEC01 therapy in DMD patients. **A** Manufacturing of DT-DEC01 begins with muscle biopsies harvested from the DMD patient and the normal donor, followed by myoblasts isolation and expansion, PKH staining and PEG-mediated fusion creating DEC cells, followed by DEC sorting, expansion, product formulation and DT-DEC01 administration to DMD patient. **B** The timeline of EMG parameters assessment of MUP duration and amplitudes in the selected muscles (deltoideus, biceps brachii, rectus femoris and gastrocnemius) of DMD patients at the scheduled visits of: *V0a* screening visit, *V0b* skeletal muscle biopsy of DMD patient and the normal donor, *V1* intraosseous DT-DEC01 administration. Active follow-up of 6 months after DT-DEC01 administration: *V2* hospital discharge day, *V3* week 1, *V4* month 1, *V5* month 3, *V6* month 6. Passive follow-up of 18 months after DT-DEC01 administration: *V7* month 12, *V8* month 18, *V9* month 24, *EMG* electromyography assessment. **C** Selected muscles of DMD patients assessed by EMG. Upper extremity: deltoideus muscle and biceps brachii; lower extremity: rectus femoris and gastrocnemius muscle. Figure created with BioRender.com

The primary objective of this pilot study was to monitor the safety of DT-DEC01 therapy. The secondary aim presented in this report was to assess therapy efficacy using standard needle EMG as the potential biomarker of DT-DEC01 personalized therapy effectiveness in DMD patients. The study design is outlined in Fig. 1A.

### Participants

Currently, three DMD patients and the donors of the skeletal muscle tissue were enrolled to the study and completed 12-month follow-up based on the data collected at the screening visit, as previously described (Heydemann et al. 2023). Briefly, the enrollment criteria for patients included DMD genetic diagnosis, corticosteroid treatment before enrollment, lack of pre-existing donor-specific antibodies, and negative serological status for infectious diseases. Each pair of participants, DMD patients (*n* = 3) and respective donors (*n* = 3), underwent standard skeletal muscle biopsy of the selected muscle (deltoideus or biceps brachii of the upper limb or vastus lateralis of the lower limb) performed under anesthesia in the hospital settings. Following biopsy, muscle tissue samples were submitted to the Polish Stem Cells Bank (Warsaw, Poland) for preparation of personalized DT-DEC01 product.

### Manufacturing of the Personalized DT-DEC01 Therapy

The established protocol of myoblast cells isolation, cell fusion and DT-DEC01 manufacturing was employed as previously described (Fig. 1A) (Heydemann and Siemionow 2023; Heydemann et al. 2023). Shortly, after the biopsy, muscle tissue samples were digested with collagenase for myoblast isolation. The cells were propagated and passaged to achieve the required cell number for the cell fusion procedure. The myoblast cells of the normal donor and the DMD patient were fluorescently labeled with PKH26 or PKH67 dyes (Sigma-Aldrich), respectively, followed by ex vivo PEG-mediated cell fusion procedure. After fusion, the created double-positive (PKH26/PKH67) chimeric DEC cells were selected via FACS MACSQuant Tyto sorter (Miltenyi Biotec). The manufacturing of patient-specific DT-DEC01 therapy was continued to achieve the dose of 2 × 10^6^ cells per kg body weight for systemic–intraosseous administration to the DMD patient (Fig. 1A).

### Systemic–Intraosseous Administration of the DT-DEC01 Therapy

Following manufacturing of the personalized DT-DEC01 therapy created via ex vivo PEG-mediated fusion of myoblasts from the normal, healthy donor and the DMD patient, DT-DEC01 cells were transported to the hospital for administration to the DMD patients.

Each of the three DMD participants received DT-DEC01 product under anesthesia via intraosseous delivery route into the bone marrow cavity of the iliac crest, as previously reported (Heydemann and Siemionow 2023; Heydemann et al. 2023). Each DT-DEC01 product contained an individually calculated number of DEC cells corresponding to a single dose of 2 × 10^6^ cells per kg of patient’s body weight. No immunosuppression was given. After administration of DT-DEC01 therapy, patients were hospitalized for 24 h and were monitored for any signs of local or systemic response that could be related to the procedure of systemic–intraosseous DT-DEC01 administration (Fig. 1A).

### Safety Assessment of the DT-DEC01 Therapy

The primary aim of this study was the safety of DT-DEC01 therapy assessed by clinical observation of the incidence and severity of therapy-related, clinically relevant abnormal findings in the vital signs, physical examination and blood testing. Safety evaluation of Adverse Events (AE) and Serious Adverse Events (SAE) was conducted from the time of skeletal muscle biopsy through the 6-month period of the active follow-up of DMD patients after DT-DEC01 therapy administration, whereas SAE were monitored continuously during the active and passive follow-up period up to 24 months. Adverse Events of Special Interest (AESI) were monitored during the first month following DT-DEC01 therapy administration.

### Preliminary Efficacy Assessment of DT-DEC01 Therapy by EMG

The EMG evaluation was performed in a standard manner by a certified neurologist, with over 20 years of experience in the assessment of DMD patients. The recordings were taken at the baseline (screening visit) and at 3, 6, and 12 months after DT-DEC01 therapy administration (Fig. 1B). Concentric needle electrodes (Neuroline Concentric, Ambu, 28 G, 30 mm) were inserted into the following muscles of the upper extremity: the right deltoideus and the right biceps brachii, and in the lower extremity: the right rectus femoris and the right gastrocnemius muscle (Fig. 1C). The EMG system (VIASYS Synergy EMG System, Medelec) was used to automatically isolate and register at least 10 MUPs from each muscle during mild and submaximal voluntary contraction for a total of over 160 MUPs recorded per patient. Next, the registered MUPs were analyzed quantitatively using the Synergy application software (Viasys Synergy, Medelec) for the duration time, the amplitudes, and the percentage of the polyphasic MUPs. The EMG rater was blinded to the results of examinations performed at 3, 6, and 12 months after DT-DEC01 therapy administration.

### Assessment of Correlation Between the MUP Duration Evaluated by EMG and the Upper Extremity Function Assessed by Functional Tests of PUL 2.0 and Grip Strength in the Selected Muscles of the Upper Extremity

The correlation analysis was performed for the outcomes assessed in the upper extremities for all three patients, regardless of the ambulation status. Accordingly, the correlation was assessed between the MUP duration values acquired by EMG in the selected muscles of the upper extremity (the deltoideus and the biceps brachii) and the upper extremity functional tests of the PUL 2.0 test and the grip strength test measured by the dynamometer at the baseline and at 3, 6, and 12 months after DT-DEC01 therapy administration.

PUL 2.0 test was assessed at 3, 6, and 12 months after DT-DEC01 therapy administration to evaluate upper extremity function as described previously (Heydemann et al. 2023; Siemionow et al. 2023). Briefly, patients were instructed to perform tasks at high (shoulder), mid (elbow), and distal (wrist and hand) level (Mayhew et al. 2013, 2020).

The grip strength was measured at 3, 6, and 12 months after DT-DEC01 therapy administration using handheld electronic dynamometer (WWEH101, Moga) as described previously (Heydemann et al. 2023; Siemionow et al. 2023). Briefly, patients performed voluntary contractions of each hand applying as much force as possible in three repetitions. Next, the grip strength results in kilograms assessed at baseline and at the follow-up visits were calculated as the percentage of results obtained at baseline.

### Statistics

The analysis for statistical significance was performed using GraphPad Prism ver. 9.5.0 software. The data are shown as mean ± SEM. The normality of the data was verified by the Shapiro–Wilk test. The statistical significance was assessed by Kruskal–Wallis test with the *P* values below 0.05 denoting significant differences. The Pearson correlation test was used to estimate correlation coefficients. The threshold for the correlation coefficient (*r*) and the *P* values were set at r of ≥ 0.7 for strong correlation (Schober et al. 2018) and for *P* of ≤ 0.05 for statistical significance.

## Results

### Study Population and DT-DEC01 Therapy

Currently, three DMD patients have completed the 12-month follow-up evaluation after receiving a single, low dose of DT-DEC01 therapy (2 × 10^6^ cells per kg) via intraosseous administration, including two ambulatory patients (Patient 1 and Patient 3) and one non-ambulatory patient (Patient 2), as per the scheduled visits (Fig. 1B).

Patient 1, who is ambulatory and was 6 years old at the time of enrollment, was diagnosed at age of 6 with genetically confirmed DMD (exon 3–12 deletion). He had been on steroid therapy for 6 months prior to study inclusion. DEC cells in the DT-DEC01 product were derived from the patient’s autologous myoblasts isolated from right vastus lateralis muscle biopsy and from normal allogeneic myoblasts derived from the vastus lateralis muscle of the donor (patient’s father).

Patient 2, who is non-ambulatory and was 15 years old at the time of enrollment, was diagnosed at age of 4 with genetically confirmed DMD (exon 48–50 deletion). He had been wheelchair-dependent since the age of 11 years and 10 months and on steroid therapy for 11 years preceding inclusion to the study. DEC cells in DT-DEC01 product were derived from the patient’s autologous myoblasts isolated from the right biceps brachii muscle biopsy and from normal allogeneic myoblasts derived from the quadriceps femoris muscle of the donor (patient’s father).

Patient 3, who is ambulatory and was 6 years old at the time of enrollment, was diagnosed at the age of 4 with genetically confirmed DMD (nonsense mutation). He had been on steroid therapy for 2 years prior to the study inclusion. DEC cells in DT-DEC01 product were derived from the patient’s autologous myoblasts isolated from the left biceps brachii muscle biopsy and from normal allogeneic myoblasts derived from the quadriceps femoris of the donor (patient’s father).

### Clinical Outcomes: Safety of a Single Dose of DT-DEC01 Therapy was Confirmed up to 22 Months After Systemic–Intraosseous Administration

According to the visits schedule, the continuous patient assessment and monitoring was taking place over the entire follow-up period after systemic–intraosseous administration of a single dose of DT-DEC01 therapy without immunosuppression. Safety of the DT-DEC01 therapy was confirmed up to 22 months (range 18–22) after systemic–intraosseous administration of DT-DEC01 as evidenced by the lack of reports on the therapy-related AE, SAE, or AESI.

### Clinical Outcomes: Preliminary Efficacy of DT-DEC01 was Confirmed by EMG Assessment at 3, 6, and 12 Months After Systemic–Intraosseous Administration

The EMG assessment of the selected muscles of the upper and lower extremities of three patients enrolled in this pilot study revealed improvements in all tested EMG parameters, including MUP duration, amplitudes, and the polyphasic MUPs. The detailed description of the collected EMG data is presented below.

### Confirmation of Increase of the MUP Duration up to 12 Months After Systemic–Intraosseous Administration of DT-DEC01 Therapy

Below is a summary of the MUP duration values in three patients enrolled to the study and assessed at the baseline (before DT-DEC01 treatment) and at 3, 6, and 12 months after intraosseous administration of a single dose (2 × 10^6^ cells/kg of body weight) of DT-DEC01 therapy.

Detailed outcomes of the EMG assessments of MUP duration including the number of MUPs analyzed in the selected muscles of the upper and lower extremities of DMD patients at each visit are summarized in Table 1.

**Table 1:** The summary of average MUP duration assessed by EMG in the selected muscles of the DMD patients at baseline and at 3, 6, and 12 months after systemic–intraosseous administration of DT-DEC01 therapy

Parameter	The average MUP duration* (ms)							
	Deltoideus	MUP (*n*)	Biceps brachii	MUP (*n*)	Rectus femoris	MUP (*n*)	Gastrocnemius	MUP (*n*)
**Patient 1**								
Baseline	3.65 ± 0.21	10	3.96 ± 0.14	10	3.27 ± 0.19	7	4.37 ± 0.25	4
Month 3	4.84 ± 0.29	10	5.83 ± 0.26	10	3.73 ± 0.14	11	3.47 ± 0.29	10
Month 6	4.18 ± 0.12	12	7.08 ± 0.76	10	4.59 ± 0.40	11	5.61 ± 0.58	11
Month 12	4.55 ± 0.30	11	3.86 ± 0.11	11	3.63 ± 0.18	12	6.66 ± 0.17	16
**Change from baseline at Month 12**	**24.5 ± 8.2%**	43	**−2.5 ± 2.8%**	41	**11.0 ± 5.4%**	41	**52.6 ± 4.0%**	41
**Patient 2**								
Baseline	2.60 ± 0.23	10	2.12 ± 0.14	10	3.16 ± 0.12	10	5.87 ± 0.27	10
Month 3	5.66 ± 0.46	11	3.86 ± 0.17	11	3.27 ± 0.12	10	4.72 ± 0.16	10
Month 6	4.96 ± 0.40	10	5.15 ± 0.28	11	3.11 ± 0.15	10	7.62 ± 0.28	10
Month 12	3.48 ± 0.24	14	5.29 ± 0.41	12	5.16 ± 0.25	12	7.82 ± 0.45	14
**Change from baseline at** **Month 12**	**33.6 ± 9.3%**	45	**149.6 ± 19.3%**	44	**63.5 ± 7.8%**	42	**33.2 ± 7.6%**	44
**Patient 3**								
Baseline	3.76 ± 0.33	10	3.49 ± 0.36	10	4.06 ± 0.34	10	4.92 ± 0.16	11
Month 3	6.21 ± 0.49	12	4.17 ± 0.30	10	6.33 ± 0.45	12	6.09 ± 0.22	12
Month 6	5.94 ± 0.34	13	5.10 ± 0.18	10	4.83 ± 0.27	12	7.15 ± 0.34	10
Month 12	6.01 ± 0.29	14	4.60 ± 0.19	13	4.52 ± 0.18	13	5.47 ± 0.27	12
**Change from baseline at** **Month 12**	**59.9 ± 7.6%**	49	**31.6 ± 5.5%**	43	**11.3 ± 4.4%**	47	**11.1 ± 5.5%**	45
**Average change from baseline at** **Month 12**	**40.5 ± 5.4%**	Total MUP 137	**60.5 ± 12.7%**	Total MUP 128	**28.1 ± 5.3%**	Total MUP 130	**33.8 ± 4.2%**	Total MUP 130

*The data are presented as mean ± SEM. A grand total of 525 MUPs were assessed

#### Patient 1 (Ambulatory, 6-Year-Old, Deletion of Exons 3–12)

**Fig. 2:**
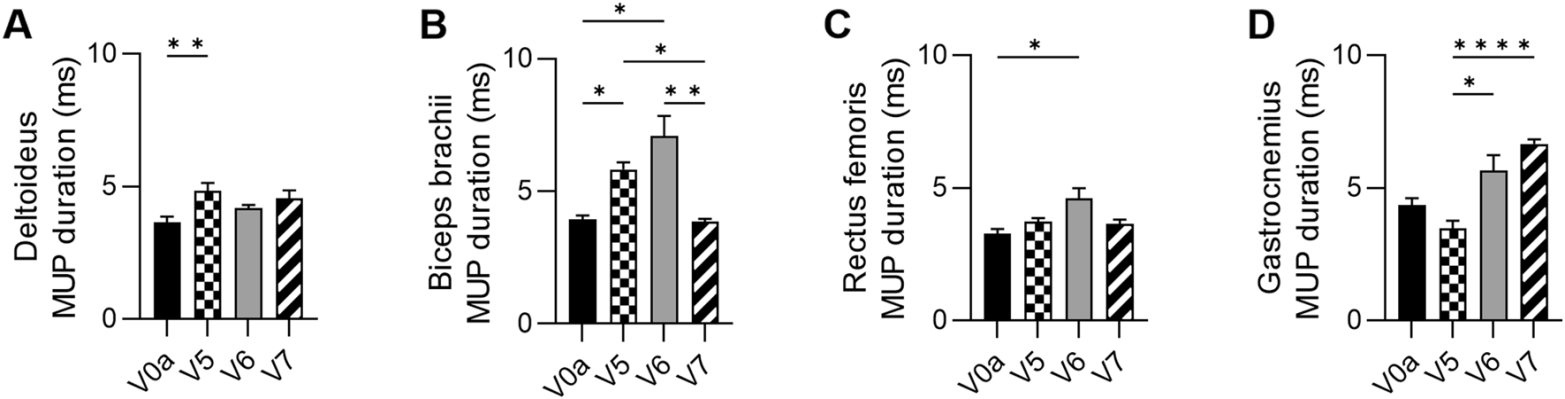
EMG outcomes assessed in Patient 1 up to 12 months after systemic–intraosseous DT-DEC01 administration. EMG assessment of average MUP duration revealed: **A** increase in the deltoideus by 24.5 ± 8.3%, **B** preservation of MUP duration in the biceps brachii (97.5 ± 2.8% of baseline duration), **C** increase in the rectus femoris by 11.0 ± 5.4% and **D** significant increase in the gastrocnemius muscle by 52.6 ± 4.0% compared to baseline. The data are expressed as mean ± SEM, and the average of 10 MUP measurements is shown. The statistical significance was assessed by Kruskal–Wallis test, with *P ≤ 0.05, **P ≤ 0.01, ***P ≤ 0.001, ****P ≤ 0.0001 denoting significant differences. *V0a* screening visit, *V5* 3-month, *V6* 6-month, *V7* 12-month visit after DT-DEC01 administration

At 3 months after DT-DEC01 administration, when compared to the baseline, EMG assessment revealed an increase in the MUP duration values: in the deltoideus increase by 32.6 ± 8.0% (*P* ≤ 0.01), in the biceps brachii by 47.5 ± 6.6% (*P* ≤ 0.05) and in the rectus femoris increase by 14.1 ± 4.1%, whereas in the gastrocnemius muscle, there was a 20.4 ± 6.5% decrease observed (Fig. 2A–D and Table 1).

At 6 months after DT-DEC01 administration, when compared to the baseline, EMG recordings of the MUP duration showed increase in all assessed muscles of both, the upper and lower extremities: in the deltoideus increase by 14.4 ± 3.4%, in the biceps brachii by 79.1 ± 19.3% (*P* ≤ 0.05), in the rectus femoris increase by 40.3 ± 12.3% (*P* ≤ 0.05) and in the gastrocnemius muscle increase by 29.7 ± 13.2% (Fig. 2A–D and Table 1).

At 12 months after DT-DEC01 administration, when compared to the baseline, EMG assessment revealed an increase in MUP duration: in the deltoideus by 24.5 ± 8.2%, in the rectus femoris by 11.0 ± 5.4% and in the gastrocnemius muscle increase by 52.6 ± 4.0%. The MUP duration in the biceps brachii muscle was preserved at the baseline level, (Fig. 2A–D and Table 1). In summary, during the 12-month assessment, there was a sustained increase compared to the baseline in MUP duration in the deltoideus, rectus femoris, and gastrocnemius muscles, while the MUP duration in the biceps brachii muscle was preserved at the baseline level.

The EMG assessment of the MUP duration in Patient 1 at the baseline and at 3, 6, and 12-month follow-up after DT-DEC01 administration is presented in Fig. 2.

#### Patient 2 (Non-ambulatory, 15-Year-Old, Deletion of Exons 48–50)

**Fig. 3:**
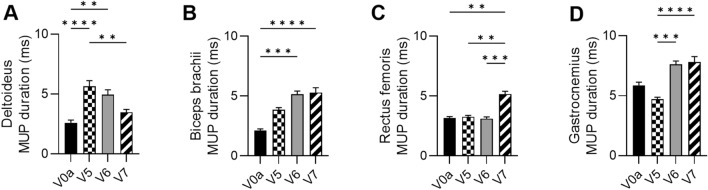
EMG outcomes assessed in Patient 2 up to 12 months after systemic–intraosseous DT-DEC01 administration. EMG assessment of average MUP duration revealed significant increase: **A** in the deltoideus by 33.6 ± 9.3%, **B** in the biceps brachii 149.6 ± 19.3%, **C** in the rectus femoris by 63.5 ± 7.8% and **D** in the gastrocnemius by 33.2 ± 7.6% compared to baseline. The data are expressed as mean ± SEM, and the average of 10 MUP measurements is shown. The statistical significance was assessed by Kruskal–Wallis test, with *P ≤ 0.05, **P ≤ 0.01, ***P ≤ 0.001, ****P ≤ 0.0001 denoting significant differences. *V0a* screening visit, *V5* 3-month, *V6* 6-months, *V7* 12-month visit after DT-DEC01 administration

At 3 months after DT-DEC01 administration, when compared to the baseline, EMG assessment revealed increase in MUP duration in muscles of the upper extremity: in the deltoideus by 117.4 ± 17.7% (*P* ≤ 0.001) and in the biceps brachii by 82.3 ± 8.1%, while the MUP duration in the lower extremity muscles was either comparable to the baseline level in the rectus femoris or was decreased by 19.6 ± 2.7% in the gastrocnemius (Fig. 3A–D and Table 1).

At 6 months after DT-DEC01 administration, when compared to the baseline, EMG assessment showed an increase in MUP duration in the upper extremity: in the deltoideus by 90.5 ± 15.5% (*P* ≤ 0.01) and in the biceps brachii by 142.9 ± 13.1%, (*P* ≤ 0.001). The MUP duration in the rectus femoris remained at baseline level. In contrast, the MUP duration in the gastrocnemius increased by 29.8 ± 4.7% (Fig. 3A–D and Table 1).

At 12 months after DT-DEC01 administration, when compared to the baseline, EMG assessment revealed an increase in MUP duration in all tested muscles of both upper and lower extremities, in the deltoideus increase by 33.6 ± 9.3%, in the biceps brachii by 149.6 ± 19.3% (*P* ≤ 0.0001), in the rectus femoris increase by 63.5 ± 7.8% (*P* ≤ 0.01) and in the gastrocnemius by 33.2 ± 7.6% (Fig. 3A–D and Table 1).

EMG assessment of MUP duration in Patient 2 at the baseline and at 3, 6, and 12-month follow-up after DT-DEC01 administration is presented in Fig. 3.

#### Patient 3 (Ambulatory, 6-Year-Old, Nonsense Mutation)

**Fig. 4:**
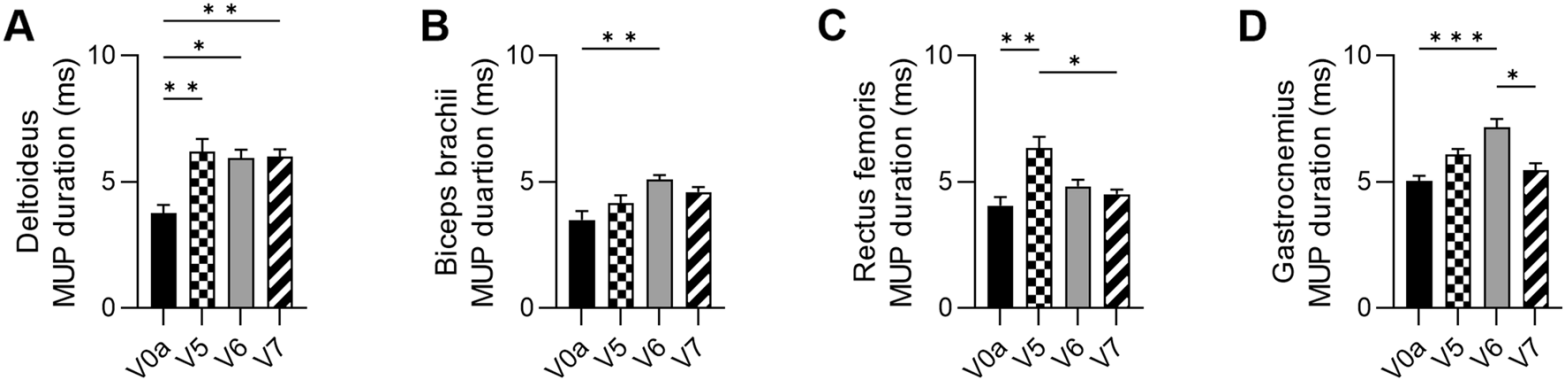
EMG outcomes assessed in Patient 3 up to 12 months after systemic–intraosseous DT-DEC01 administration. EMG assessment of average MUP duration revealed: **A** significant increase in the deltoideus by 59.9 ± 7.6%, **B** increase in the biceps brachii by 31.6 ± 5.5%, **C** increase in the rectus femoris by 11.3 ± 4.4% and **D** increase in the gastrocnemius by 11.1 ± 5.5% compared to baseline. The data are expressed as mean ± SEM, and the average of 10 MUP measurements is shown. The statistical significance was assessed by Kruskal–Wallis test, with *P ≤ 0.05, **P ≤ 0.01, ***P ≤ 0.001, ****P ≤ 0.0001 denoting significant differences. *V0a* screening visit, *V5* 3 months, *V6* 6 months, *V7* 12 months of visit after DT-DEC01 administration

At 3 months after DT-DEC01 administration, when compared to the baseline, EMG assessment revealed increase in MUP duration in all tested muscles of both, the upper and lower extremities: in the deltoideus by 65.3 ± 13.1% (*P* ≤ 0.01), in the biceps brachii by 19.2 ± 8.6%, in the rectus femoris by 55.9 ± 11.1% (*P* ≤ 0.01) and in the gastrocnemius by 23.6 ± 4.5% (Fig. 4A–D and Table 1).

At 6 months after DT-DEC01 administration, when compared to the baseline, EMG assessment showed an increase in MUP duration in all tested muscles: in the deltoideus by 58.0 ± 9.1% (*P* ≤ 0.05), in the biceps brachii an increase by 45.9 ± 5.1% (*P* ≤ 0.01), in the rectus femoris by 18.9 ± 6.6% and in the gastrocnemius an increase by 45.3 ± 6.9% (Fig. 4A–D and Table 1).

At 12 months after DT-DEC01 administration, when compared to the baseline, EMG assessment revealed an increase in MUP duration in all tested muscles of both upper and lower extremities: in the deltoideus increase by 59.9 ± 7.6% (*P* ≤ 0.01), in the biceps brachii by 31.6 ± 5.5%, in the rectus femoris by 11.3 ± 4.4% and in the gastrocnemius muscle an increase by 11.1 ± 5.5% (Fig. 4A–D and Table 1).

EMG assessment of MUP duration in Patient 3 at the baseline and at 3, 6, and 12-month follow-up after intraosseous DT-DEC01 administration is presented in Fig. 4.

### Confirmation of Improvement in Tested EMG Parameters in Three DMD Patients at 12 Months After Systemic–Intraosseous Administration of DT-DEC01 Therapy

#### Confirmation of the Increase in the MUP Duration Assessed by the EMG in Three DMD Patients

**Fig. 5:**
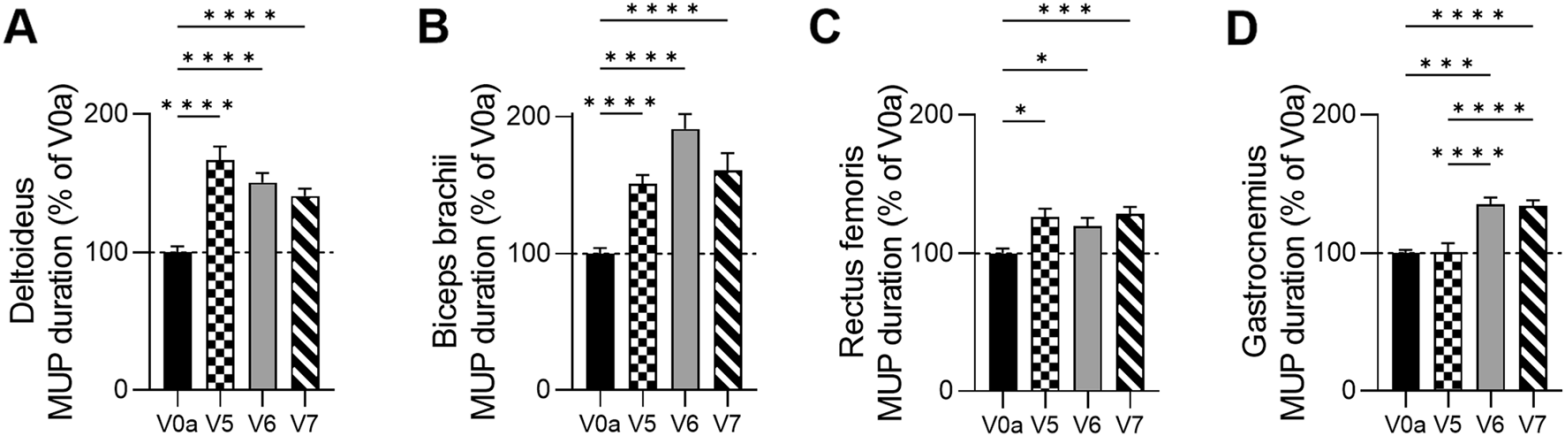
The summary of the Motor Unit Potentials (MUP) duration assessed by EMG in three DMD patients at baseline and up to 12 months after systemic–intraosseous administration of DT-DEC01 therapy. The assessment of average MUP duration in three patients revealed a significant increase in all tested muscles: **A** in the deltoideus by 40.5 ± 5.4%, **B** in the biceps brachii by 60.5 ± 12.7%, **C** in the rectus femoris by 28.1 ± 5.3% and **D** in the gastrocnemius by 33.8 ± 4.2%. The data are expressed as mean ± SEM, and the average of 33 MUP measurements is shown. The statistical significance was assessed by Kruskal–Wallis test, with *P ≤ 0.05, **P ≤ 0.01, ***P ≤ 0.001, ****P ≤ 0.0001 denoting significant differences. *V0a* screening visit, *V5* 3 months, *V6* 6 months, *V7* 12 months of visit after DT-DEC01 administration

A comparative analysis of the MUP duration was performed in both, the ambulatory and non-ambulatory patients at the screening visit, the baseline before DT-DEC01 treatment and at 3, 6, and 12 months after systemic—intraosseous administration of a single dose (2 × 10^6^ cells/kg of body weight) of DT-DEC01 therapy and revealed significant increase in the MUP duration in all tested muscles (Fig. 5 and Table 1).

At 3 months after DT-DEC01 administration, when compared to the baseline, assessment of EMG recordings in three patients revealed: an increase in the average MUP duration in the deltoideus by 66.4 ± 10.1% (*P* ≤ 0.0001), in the biceps brachii by 50.7 ± 6.5% (*P* ≤ 0.0001) and in the rectus femoris increase by 26.1 ± 5.9% (*P* ≤ 0.05). In the gastrocnemius average MUP, duration was maintained at the baseline level (Fig. 5A–D and Table 1).

At 6 months after DT-DEC01 administration, when compared to the baseline, the EMG assessment in three patients revealed an increase in the average MUP duration in all assessed selected skeletal muscles of the upper and lower extremities. For the upper extremity, the deltoideus showed an increase by 50.2 ± 7.1% (*P* ≤ 0.0001) and the biceps brachii revealed an increase by 91.1 ± 10.7% (*P* ≤ 0.0001). In the lower extremities, the rectus femoris displayed an increase by 19.9 ± 5.6% (*P* ≤ 0.05) and in the gastrocnemius, there was an increase of 34.9 ± 5.2% (Fig. 5A–D and Table 1).

At 12 months after DT-DEC01 administration, when compared to the baseline, the EMG assessment in three patients revealed an increase in the average MUP duration in all tested skeletal muscles. The deltoideus showed an average increase by 40.5 ± 5.4% (*P* ≤ 0.0001), the biceps brachii increase by 60.5 ± 12.7% (*P* ≤ 0.0001), the rectus femoris revealed increase by 28.1 ± 5.3% (*P* ≤ 0.001), and the gastrocnemius increase by 33.8 ± 4.2% (*P* ≤ 0.0001) (Fig. 5A–D and Table 1).

The summary of the average MUP duration assessed by EMG in three patients at the baseline and at 3-, 6-, and 12-month follow-up after intraosseous DT-DEC01 administration is presented in Fig. 5.

#### Confirmation of the Increase in the MUP Amplitudes Assessed by EMG in Three DMD Patients

**Fig. 6:**
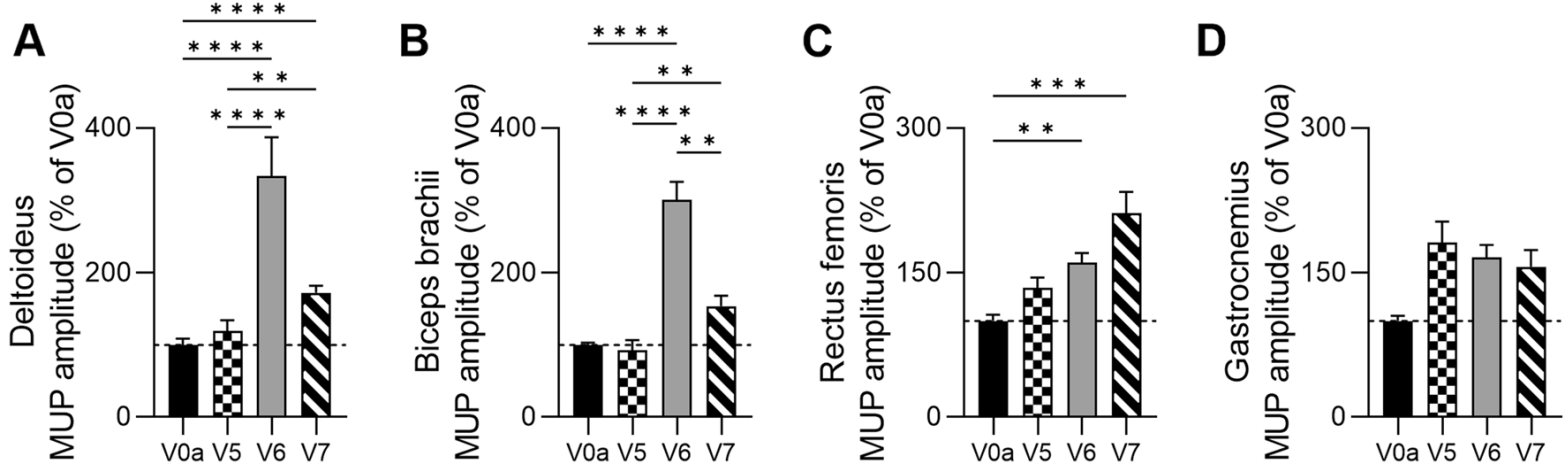
Summary of the motor unit potentials (MUP) amplitudes assessed by EMG in three DMD patients up to 12 months after systemic–intraosseous DT-DT-DEC01 therapy administration. Assessment of average MUP amplitude in three patients revealed increase in all tested muscles: **A** significant increase in deltoideus by 72.2 ± 9.8%, **B** increase in biceps brachii by 53.9 ± 14.1%, **C** significant increase in rectus femoris by 111.5 ± 21.9% and **D** increase in gastrocnemius by 56.2 ± 17.4%. The data are expressed as mean ± SEM, and the average of 33 MUP measurements is shown. The statistical significance was assessed by Kruskal–Wallis test, with *P ≤ 0.05, **P ≤ 0.01, ***P ≤ 0.001, ****P ≤ 0.0001 denoting significant differences. *V0a* screening visit, *V5* 3-month, *V6* 6-month, *V7* 12-month visit after DT-DEC01 administration

A comparative analysis of the MUP amplitudes in three DMD patients, assessed by EMG at the screening visit—the baseline, and at 3, 6, and 12 months after DT-DEC01 therapy administration, revealed an increase in the average MUP amplitude values in all tested muscles (Fig. 6 and Table 2).

**Table 2:** The summary of average MUP amplitude recordings assessed by EMG in the selected muscles of the DMD patients at baseline and at 3, 6, and 12 months after systemic–intraosseous administration of DT-DEC01 therapy

Parameter	The average MUP amplitude* (µV)							
	Deltoideus	MUP (*n*)	Biceps brachii	MUP (*n*)	Rectus femoris	MUP (*n*)	Gastrocnemius	MUP (*n*)
**Patient 1**								
Baseline	684 ± 69	10	811 ± 18	10	298 ± 40	7	883 ± 34	4
Month 3	911 ± 81	10	155 ± 5	10	225 ± 23	11	238 ± 21	10
Month 6	1087 ± 55	12	1310 ± 195	10	620 ± 37	11	678 ± 62	11
Month 12	826 ± 40	11	831 ± 48	11	645 ± 33	12	605 ± 117	16
**Change from baseline at** **Month 12**	**20.8 ± 5.8%**	43	**2.5 ± 5.9%**	41	**116.5 ± 10.9%**	41	**−31.6 ± 13.3%**	41
**Patient 2**								
Baseline	1408 ± 282	10	1667 ± 70	10	2234 ± 115	10	970 ± 128	10
Month 3	613 ± 52	11	1101 ± 52	11	3771 ± 32	10	2936 ± 183	10
Month 6	2652 ± 201	10	6444 ± 463	11	3397 ± 359	10	1955 ± 86	10
Month 12	2250 ± 176	14	2101 ± 257	12	1078 ± 99	12	1297 ± 142	14
**Change from baseline at** **Month 12**	**59.7 ± 12.5%**	45	**26.1 ± 15.4%**	44	**−51.7 ± 4.4%**	42	**33.7 ± 14.6%**	44
**Patient 3**								
Baseline	540 ± 77	10	928 ± 68	10	916 ± 133	10	723 ± 20	11
Month 3	985 ± 158	12	1818 ± 76	10	1459 ± 188	12	1517 ± 97	12
Month 6	3279 ± 582	13	3204 ± 345	10	1133 ± 110	12	1584 ± 112	10
Month 12	1216 ± 80	14	2050 ± 256	13	3275 ± 114	13	2113 ± 173	12
**Change from baseline at Month 12**	1**25.1 ± 14.8%**	**49**	**121.0 ± 27.6%**	**43**	**257.6 ± 12.4%**	**47**	**192.1 ± 23.9%**	**45**
**Average change from baseline at** M**onth 12**	**72.2 ± 9.8%**	Total MUP 137	**53.9 ± 14.1%**	Total MUP 128	**111.5 ± 21.9%**	Total MUP 130	**56.2 ± 17.4%**	Total MUP 130

*The data are presented as mean ± SEM. A grand total of 525 MUPs were assessed

At 3 months after DT-DEC01 administration, when compared to the baseline, the EMG assessments in the three DMD patients showed an increase in the average MUP amplitude values in the deltoideus by 19.3 ± 14.8%, while the MUP amplitudes in the biceps brachii were maintained at baseline level. The lower extremity muscles showed an increase in the MUP amplitude values: in the rectus femoris MUP amplitude increased by 34.6 ± 10.7%, and in the gastrocnemius muscle by 81.6 ± 21.3% (Fig. 6A–D and Table 2).

At 6 months after DT-DEC01 administration, when compared to the baseline, the EMG assessments in the three DMD patients revealed an increase in the average MUP amplitude values in all tested muscles of both upper and lower extremities: in the deltoideus increase by 233.7 ± 53.3%, in the biceps brachii by 200.8 ± 24.5%, in the rectus femoris increase by 60.5 ± 9.8% and in the gastrocnemius by 65.8 ± 13.3% (Fig. 6A–D and Table 2).

At 12 months after DT-DEC01 administration, when compared to the baseline, the EMG assessments in the three DMD patients revealed an increase in the average MUP amplitude values in all tested muscles: in the deltoideus increase by 72.2 ± 9.8%, in the biceps brachii by 53.9 ± 14.1%, in the rectus femoris by 111.5 ± 21.9% and in the gastrocnemius by 59.2 ± 17.4% (Fig. 6A–D and Table 2).

The summary of the average MUP amplitudes assessed by EMG at the baseline and at 3, 6, and 12-month follow-up after DT-DEC01 administration is presented in Fig. 6.

#### Confirmation of the Increase in the Percentage of Polyphasic MUPs Assessed by EMG in Three DMD Patients

**Fig. 7:**
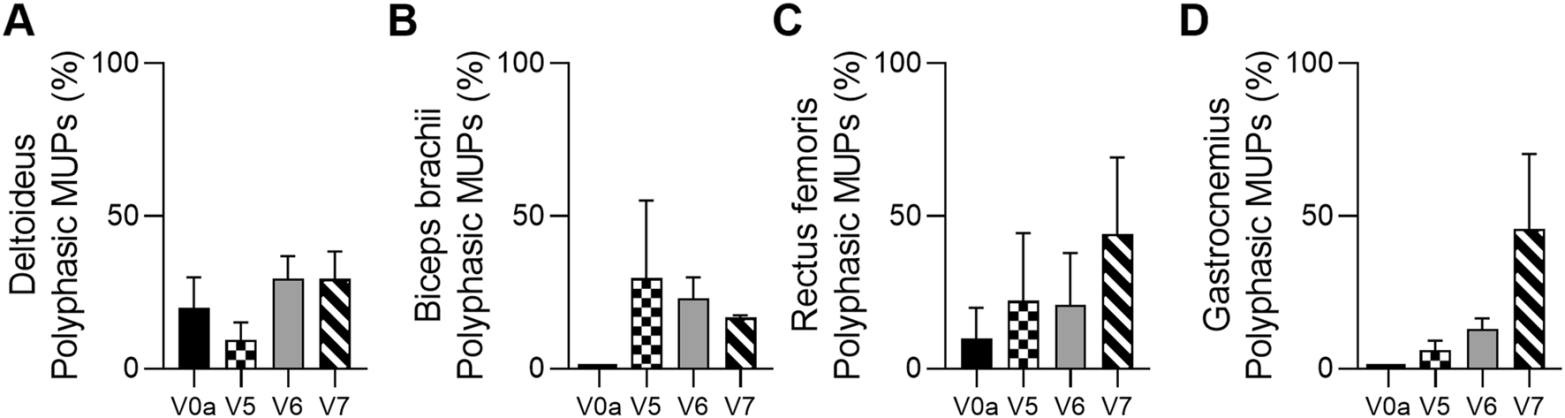
The summary of the polyphasic Motor Unit Potentials (MUP) assessed by EMG in the three DMD patients from the baseline up to 12 months after systemic–intraosseous administration of the DT-DEC01 therapy. EMG assessment of the polyphasic MUPs in the three patients revealed increase in the percentage of the polyphasic MUPs when compared to the baseline values in all tested muscles: **A** in the deltoideus from 20.0% to 29.5%, **B** in the biceps brachii from 0.0% to 16.8%, **C** in the rectus femoris from 10.0% to 44.2% and **D** in the gastrocnemius from 0.0% to 45.8%. The data are expressed as mean ± SEM, and the average of 3 MUP measurements is shown. *V0a* screening visit, *V5* 3-month, *V6* 6-month, *V7* 12-month visit post-DT-DEC01 administration

A comparative analysis of the percentage of the polyphasic MUPs was conducted in three DMD patients at the screening visit - the baseline and at 3, 6, and 12 months after DT-DEC01 therapy administration and revealed an increase in the percentage of the polyphasic MUPs (Fig. 7).

At 3 months after DT-DEC01 administration, when compared to the baseline, the analysis of EMG assessments in the three DMD patients revealed a decrease in the average percentage of polyphasic MUPs in the deltoideus from 20.0 ± 10.0% observed before treatment to 9.4 ± 5.8% of MUPs assessed at 3 months. In biceps brachii, the polyphasic MUPs were absent at baseline, at the 3-month assessment, they accounted for 29.7 ± 25.3% of all MUPs. In the rectus femoris, the percentage of polyphasic MUPs increased from 10.0 ± 10.0% to 22.2 ± 22.2%, in the gastrocnemius, polyphasic MUPs were not observed at baseline. However, they increased to 6.1 ± 3.1% of the measured MUPs at 3-month assessment (Fig. 7A–D).

At 6 months after DT-DEC01 administration, when compared to the baseline, the EMG assessments in three DMD patients revealed an increase in the polyphasic MUPs percentage in all tested muscles of both upper and lower extremities. In the deltoideus, the polyphasic MUPs increased to 29.6 ± 7.3% and in the biceps brachii, the polyphasic MUPs increased to 23.0 ± 7.0% of the MUPs assessed. In the rectus femoris, polyphasic MUPs percentage increased to 20.9 ± 17.0%, while in the gastrocnemius to 13.0 ± 3.5% (Fig. 7A–D).

At 12 months after DT-DEC01 administration, when compared to the baseline, the EMG assessments in the three DMD patients revealed an increase of the polyphasic MUPs percentage in all assessed muscles. In the deltoideus, polyphasic MUPs increased to 29.5 ± 9.0% and in the biceps brachii, the polyphasic MUPs increased to 16.8 ± 0.8% of the assessed MUPs. In the rectus femoris, polyphasic MUPs percentage increased to 44.2 ± 24.9% and in the gastrocnemius, the polyphasic MUPs percentage increased to 45.8 ± 24.0% (Fig. 7A–D).

#### Confirmation of a Correlation between MUP Duration, Assessed by EMG, and Functional Outcomes of the Upper Extremity Assessed by the PUL 2.0 Test and the Grip Strength Measured by Dynamometer

**Fig. 8:**
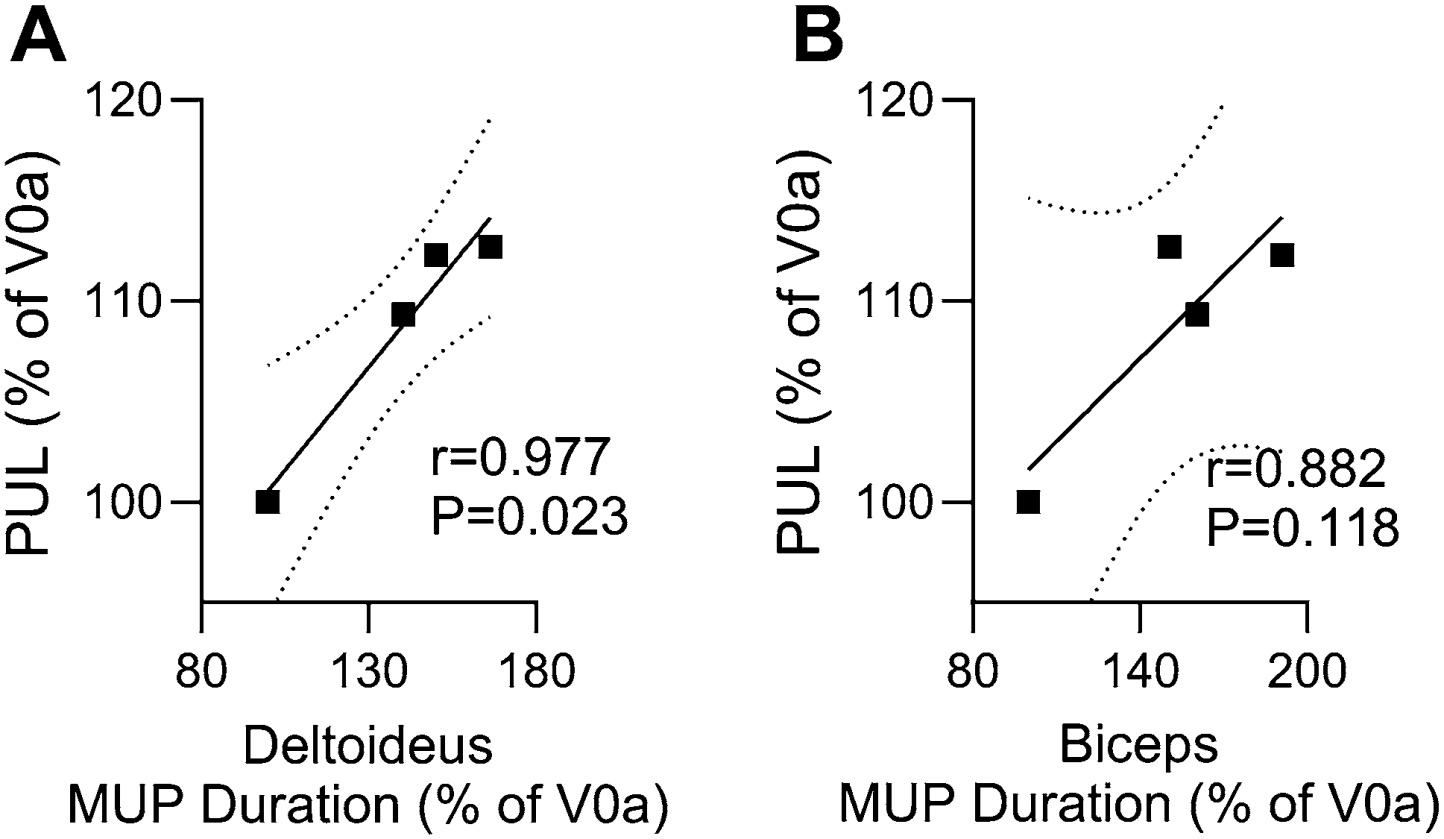
The correlation between Motor Unit Potentials (MUP) duration, assessed by EMG in the deltoideus and the biceps brachii, and upper extremity functional test of PUL 2.0 in three DMD patients from baseline up to 12 months after systemic–intraosseous administration of the DT-DEC01 therapy. The correlation analysis between MUP duration and PUL 2.0 test confirmed: **A** a strong and significant correlation between the increase in MUP duration recorded in the deltoideus muscle and the improved functional outcomes assessed by the PUL test (correlation coefficient *r* = 0.977, *P* = 0.023), **B** a strong correlation between the increase in MUP duration recorded in the biceps brachii muscle and the improved functional outcomes assessed by the PUL test (correlation coefficient *r* = 0.882; *P* = 0.118) up to the 12 months after DT-DEC01 therapy administration. The Pearson correlation coefficient was used to assess the correlation. Square points indicate correlation of PUL 2.0 outcomes and MUP duration in (**A**) deltoideus muscle and in (**B**) biceps brachii. Linear regression is presented with solid line for (**A)** deltoideus muscle correlation (*Y* = 0.2045 × *X* + 80.10) and for (**B)** biceps brachii correlation (*Y* = 0.1379 × *X* + 87.82) and with 95% confidence interval indicated by dotted line. *V0a* screening visit, *V5* 3-month, *V6* 6-month, *V7* 12-month visit after DT-DEC01 administration

**Fig. 9:**
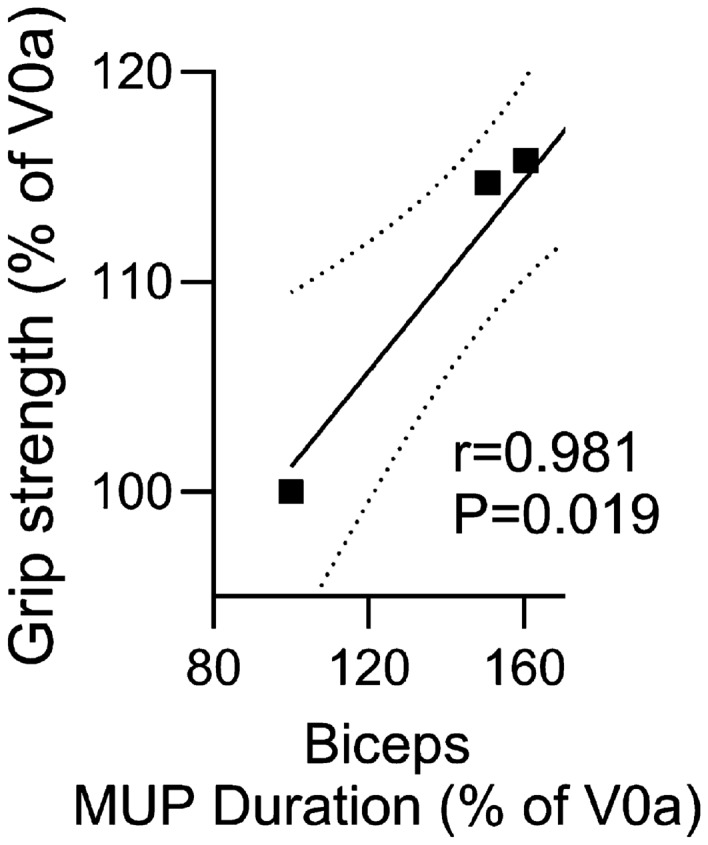
The correlation between motor unit potentials (MUP) duration assessed by EMG in the biceps brachii, and upper extremity functional test of grip strength, measured by dynamometer in three DMD patients from baseline up to 12 months after systemic–intraosseous administration of the DT-DEC01 therapy. The correlation analysis confirmed a strong and significant correlation between the increased MUP duration in the biceps brachii muscle and the improved functional outcomes of upper extremity assessed by grip strength (correlation coefficient *r* = 0.981, *P* = 0.019) throughout the 12 months of follow-up period after DT-DEC01 therapy administration. The Pearson correlation coefficient was used to assess the correlation. Square points indicate correlation of grip strength outcomes and MUP duration in biceps brachii. Linear regression is presented with a solid line (*Y* = 0.2276 × *X* + 78.40) with 95% confidence interval indicated by dotted line. *V0a* screening visit, *V5* 3-month, *V6* 6-month, *V7* 12-month visit after DT-DEC01 administration

A comparative analysis was conducted to assess the correlation between MUP duration, recorded in the deltoideus and biceps brachii muscles by EMG, and the functional outcomes assessed by the PUL 2.0 test and grip strength test in three DMD patients both, ambulatory and non-ambulatory, at the screening visit (baseline) before DT-DEC01 therapy and at 3, 6 and 12 months after DT-DEC01 therapy administration. The results revealed a strong correlation between MUP duration and the functional tests of PUL 2.0 (Fig. 8) and grip strength (Fig. 9).

At 12 months after DT-DEC01 administration, the EMG recordings in the selected muscles of the upper extremity (deltoideus and biceps brachii) assessed in three DMD patients confirmed an increase in MUP duration in the deltoideus muscle (by 40.5% compared to baseline), which revealed a significant and strong correlation with the improvement of functional outcomes assessed by the PUL 2.0 test (by 9.3%); Pearson correlation analysis: *r* = 0.977 and *P* = 0.023 (Fig. 8A).

Furthermore, an increase in MUP duration recorded in the biceps brachii muscle (by 60.5%) confirmed a strong correlation with the functional outcomes of the upper extremity assessed by the PUL 2.0 test; Pearson correlation analysis: *r* = 0.882 and *P* = 0.118 (Fig. 8B).

At 12 months after DT-DEC01 administration, the EMG assessment of the biceps brachii muscle in three DMD patients confirmed an increase in MUP duration (by 60.5%), which revealed a significant and strong correlation with the improvement of the functional outcomes assessed by grip strength, which showed an increase of 15.7%; Pearson correlation analysis*:* r = 0.981, *P* = 0.019 (Fig. 9).

## Discussion

DMD is an X-linked, progressive and lethal disease, caused by mutations in the dystrophin gene, resulting in muscle degeneration, wasting, and weakness affecting skeletal, cardiac, and respiratory muscles. The progressive muscle degradation and inadequate regeneration results in the development of chronic inflammation, fibrosis, and fat deposition, which restrict the normal functioning of the affected muscles (Muir et al. 2016; Strehle and Straub 2015). This leads to the loss of DMD patients’ mobility and development of cardiomyopathy, and deterioration of respiratory function, ultimately resulting in the premature death of DMD patients (Bushby et al. 2010). Despite scientific efforts and different therapeutic approaches, there is currently no cure for DMD patients (Himič and Davies 2021; Yao et al. 2021). To address this need, we have introduced a novel myoblast-based DT-DEC01 therapy of DEC cells created by the fusion of human myoblasts derived from normal (allogeneic) and DMD-affected (autologous) donors (Heydemann and Siemionow 2023; Heydemann et al. 2023; Siemionow et al. 2023).

The safety and the long-term efficacy of DEC cells have been confirmed in preclinical studies in the *mdx* mouse models of DMD, assessed after systemic–intraosseous administration of DEC (Heydemann and Siemionow 2023; Siemionow et al. 2018a, b, 2019, 2022a, b). Moreover, we have recently reported preliminary outcomes of the first-in-human pilot study on the DT-DEC01 therapy, which confirmed both its safety and preliminary efficacy up to 6 and 12 months after systemic–intraosseous administration (Heydemann et al. 2023; Siemionow et al. 2023). As the primary goal of the study was to assess the long-term safety of DT-DEC01 therapy, we continued to evaluate and monitor DMD patients in this study and confirmed the safety of DT-DEC01 therapy by the lack of therapy-related Adverse Events (AE) or Serious Adverse Events (SAE) up to 22 months following systemic–intraosseous DT-DEC01 administration.

The current study assessed the safety and preliminary efficacy of DT-DEC01 therapy in 6–15-year-old boys (*n* = 3) with genetically confirmed DMD. EMG assessment of selected muscles of the upper (deltoideus, biceps brachii) and lower (rectus femoris and gastrocnemius) extremities at the screening visit before DT-DEC01 treatment and at 3, 6, and 12 months following systemic–intraosseous administration of a single low dose of DT-DEC01 therapy. The study received Bioethics Committee approval (no. 46/2019), and no immunosuppression was administered.

The EMG assessment of selected muscles in both ambulatory and non-ambulatory patients confirmed the preliminary efficacy of DT-DEC01 therapy. Specifically, at 12 months post-treatment, there was an increase in MUP duration, amplitudes, and polyphasic MUPs. This study further validates the use of EMG as a reliable and objective biomarker for functional assessment in DMD patients after intraosseous administration of the novel DT-DEC01 therapy.

When introducing new therapeutic approaches in the rare diseases such as DMD, it is essential to assess functional outcomes with the battery of standard tests which are reliable and reproducible. This allows the results to be compared with the outcomes of different treatments applied to the same patient population.

The 6MWT and timed tasks of the NSAA test are well-accepted evaluations for ambulatory DMD patients, while the PUL is an example of a test accepted for the evaluation of non-ambulatory patients (Goemans et al. 2013; Mazzone et al. 2009, 2010, 2013; McDonald et al. 2010, 2013, 2022). However, the major challenge encountered when applying these tests in DMD patients is the fact that they are all dependent on the patient’s health on the day of testing, mood, and willingness to perform the requested tasks. Therefore, these tests cannot be considered fully objective.

In addition to the functional tests, dystrophin expression is assessed by Western Blot (WB) analysis in the samples taken from muscle biopsies of the treated DMD patients.

However, as these biopsies require anesthesia, several concerns must be addressed regarding the clinical scenario for WB assessment (van den Bersselaar et al. 2022). Clinical studies testing new gene therapies have reported WB data that confirm varying levels of dystrophin expression in muscle biopsy samples. However, these findings do not always correlate with functional improvements assessed through standard functional tests (Clemens et al. 2020, 2022; Deng et al. 2022; Duan 2018; Elangkovan and Dickson 2021; Kesselheim and Avorn 2016; Kinane et al. 2018; Mendell et al. 2016, 2020; Servais et al. 2022; Shimizu-Motohashi et al. 2018, 2019).

These statements are supported by a recent FDA report, posted regarding the micro-dystrophin study, where FDA has reservations about the lack of substantial evidence demonstrating a pharmacological impact of SRP-9001 through the dystrophin expression proposed as the surrogate biomarker. It should be noted that the study results did not show a clear association between the expression of micro-dystrophin protein and changes in the NSAA total scores as summarized: “That study demonstrated no statistically significant difference in change in NSAA scores at Week 48 between subjects who received SRP-9001 compared with those who received placebo, despite the demonstration of Sarepta’s micro-dystrophin expression at Week 12” (FDA Briefing Document BLA# 125,781/00 2023).

Moreover, several investigators have reported challenges and reliability concerns when performing immunoblots on dystrophin, which is one of the largest proteins (Aartsma-Rus et al. 2019; Anthony et al. 2014). In addition to technical challenges, there are also safety concerns, as muscle biopsies must be taken under anesthesia, which may lead to anesthesia-related complications in DMD patients (Birnkrant et al. 2007; Gurunathan et al. 2019; Hayes et al. 2007; Hemphill et al. 2019; Horikoshi et al. 2021; Muenster et al. 2012; Segura et al. 2013; van den Bersselaar et al. 2022; Yemen and Mcclain 2006). Therefore, we have searched for alternative tests that are safer, more reliable and independent of the patient’s influence on the performed tasks. One of the well-accepted methods used for evaluating DMD patients is EMG (Derry et al. 2012; Klimczak et al. 2020; Ropars et al. 2016; Szmidt-Sałkowskaet al. 2015; Verma et al. 2017).

EMG is known as an objective electrophysiological biomarker of muscle fiber function and has been universally accepted to study muscular dystrophies (Derry et al. 2012; Klimczak et al. 2020; Ropars et al. 2016; Rowinska-Marcinska et al. 2005; Szmidt-Sałkowska et al. 2015; Verma et al. 2017; Zalewska et al. 2013).

In DMD patients, EMG assessments are typically evaluated using either surface or needle electrodes (Janssen et al. 2020; Lobo-Prat et al. 2017; Nizamis et al. 2020; Zalewska et al. 2013). Surface EMG is commonly used in kinesiology to assess the functional abilities of the lower (Frigo and Crenna 2009; Ropars et al. 2016; Vandekerckhove et al. 2020) and upper extremities (Janssen et al. 2020; Trost et al. 2021) of DMD patients. However, it does not allow for a quantitative assessment of changes in single motor units in DMD-affected muscles. In contrast, needle EMG provides information on the severity of involvement of single motor units in various muscular dystrophies. Given the importance of an objective and patient-independent assessment of the efficacy of our novel DT-DEC01 therapy, we used needle EMG as a minimally invasive, quantitative, and objective method to evaluate the restoration of skeletal muscle activity and function in DMD patients after systemic–intraosseous administration of DT-DEC01 therapy (Derry et al. 2012; Heydemann et al. 2023; Klimczak et al. 2020; Ropars et al. 2016; Szmidt-Sałkowska et al. 2015; Verma et al. 2017).

We have previously reported the usefulness of EMG assessment in our pilot clinical study, where standard EMG parameters of MUPs and amplitudes were evaluated over a 6-month and 12-month follow-up period. The results revealed progressive improvements in these electrophysiological biomarkers in selected muscles of both ambulatory and non-ambulatory patients (Heydemann et al. 2023; Siemionow et al. 2023).

We continued the pilot study to confirm the efficacy of DT-DEC01 therapy using standard protocols (Derry et al. 2012; Klimczak et al. 2020; Paganoni and Amato 2013; Preston and Shapiro 2013; Ropars et al. 2016; Szmidt-Sałkowska et al. 2015; Verma et al. 2017) of needle EMG assessments up to 12 months after systemic–intraosseous administration of a single dose of DT-DEC01 to DMD patients. The results showed a significant increase in both MUP duration and amplitudes in the assessed muscles of both ambulatory and non-ambulatory DMD patients at 12 months. EMG parameters suggest that DT-DEC01 administration triggers an active, regenerative process in the muscles of DMD patients resulting in increased muscle fiber volume, leading to longer MUP duration and higher MUP amplitudes (Preston and Shapiro 2013).

Accurate interpretation of EMG assessments requires experienced neurologist and consideration of factors that influence MUP amplitudes, such as the distance of the needle from the depolarizing muscle fibers in the tested muscles and the synchronicity of the potentials. Therefore, EMG assessments in our study were performed by an established neurologist with over 20 years of experience in the evaluation of DMD patients.

The duration of the MUP (also called the motor unit action potential or MUAP) is determined by the depolarization of many muscle fibers that constitute the given motor unit with the terminal nerve branch. Comparably to the amplitude, MUP duration reflects the number of functional muscle fibers but, unlike the amplitude, is not influenced by the distance of the needle from the firing muscle fibers. Therefore, regenerating motor units which increase in size, show an increase in MUP durations (Preston and Shapiro 2013).

The amplitude of the MUP reflects mainly the volume of the motor unit and, in our study, is an indicator of the expected direction of changes within the muscles assessed after DT-DEC01 therapy. However, it is considered a less reliable parameter due to its dependence on the distance of the needle from the muscle fibers (needle position) and the probability of activating the exact unit by the patient during the actual examination. This can lead to variability of the recorded amplitudes.

In contrast, the duration of MUP reflects the time needed for the depolarization of muscle fibers in the given motor unit, which correlates with the number of functional muscle fibers and is less influenced by external factors such as needle position. Therefore, it is considered a more reliable parameter for evaluating muscle function in DMD patients (Preston and Shapiro 2013).

Remodeling of muscle fibers with more heterogeneity in electrical conduction leads to an increase in the number of MUP turns and subsequently raises the percentage of polyphasic MUP. In healthy individuals, there are usually less than 15% of polyphasic MUPs in most muscles (Crone and Krarup 2013). However, in certain conditions such as muscle denervation or reinnervation, muscle fiber remodeling can lead, at least temporarily, to an increase in the number of polyphasic MUPs.

When the motor unit shows atrophy due to underlying dystrophy, polyphasic MUPs increase, while MUP amplitudes and duration typically decrease. Considering the collected data, we hypothesize that, in response to DT-DEC01 therapy administration, an increase in polyphasic MUPs, accompanied by increase in both, MUP amplitudes and duration, may indicate that the motor unit begins to expand/rebuild. At that time, its surface may be folded or grow in an irregular spatial manner giving rise to multiple turns of the electrical vector and subsequently leading to the polyphasic MUP character. When the expansion / reconstruction stimulated by the therapy reaches its endpoint, its surface is “smoothed out” and the percentage of polyphasic MUP decreases. All the expected phenomena in the treated muscle, namely regrowth/regeneration, as well as the expected self-limitation of the organ/tissue repair should give rise to the above-described electrical presentation, according to the principles of EMG described by others (Buchthal and Pinelli 1953; Buchthal 1970) which makes our hypothesis highly plausible.

Therefore, the increased number of polyphasic MUPs with concomitant increase in MUP duration indicates that needle EMG could be regarded as an objective, minimally invasive and safer method of assessment of the treatment efficacy reducing the need for histopathological examination based on muscle biopsy taken from DMD patients under anesthesia.

Our study provides evidence of the benefits of DT-DEC01 therapy on muscle activity in DMD patients and underscores the validity of using EMG assessment as an alternative biomarker. The observed changes in EMG parameters may reflect an active process occurring in the muscles of DMD patients after administration of the DT-DEC01 therapy, indicating an increase in the volume of muscle fibers, resulting in longer MUP duration and higher amplitudes as well as an increase in the percentage of polyphasic MUPs. This may indicate an electrophysiological sign of a muscle response to DT-DEC01 therapy through the regrowth and remodeling of shape and volume of the muscle fibers in the muscles affected by DMD.

When interpreting the EMG results, it is important to emphasize that our pilot study included DMD patients of age 5–18 years old, representing different stages of the disease and different ambulatory statuses, including both ambulatory and non-ambulatory patients. Due to the diversity of mutations in the dystrophin gene, the course of DMD varies among patients. The initial effects of muscle wasting are typically observed in the lower limbs, while the upper limbs retain their function for a longer duration (Mayhew et al. 2013). Furthermore, muscle weakness typically exhibits a progression from the proximal to the distal areas (Mayhew et al. 2020).

Therefore, when performing EMG of different muscles groups, it is important to remember that due to the progressive nature of the disease, there will be differences in the EMG results assessed in different muscles of the same patient. Thus, it cannot be expected that different muscle groups affected by dystrophic changes at various stages of the disease will respond in the same manner during functional evaluations or EMG testing.

Considering these differences, our study confirms a strong clinical correlation between the increased MUP duration recorded by EMG in the deltoideus and biceps brachii muscles and the improvement in functional outcomes of the upper extremity assessed by the PUL 2.0 test and grip strength measured by the dynamometer over the 12-month period after DT-DEC01 therapy administration. The improvement of clinical outcomes assessed in the upper extremities through functional tests, correlating with the EMG results revealing an increase in MUP duration in the selected muscles of the upper extremity, supports the validity of EMG assessment as a sensitive biomarker of the muscle response to DEC therapy in both, ambulatory and non-ambulatory patients.

Several trials evaluating new treatments for DMD do not have a control/placebo group or use open-label designs (Acibadem University 2014; Capricor Inc. 2023; Clemens et al. 2020; Komaki et al. 2020; Mah et al. 2022; Pfizer 2023; ReveraGen BioPharma, Inc. 2021; Sarepta Therapeutics, Inc. 2020, 2023; Solid Biosciences Inc. 2023; Stem Cells Arabia 2020). However, there is a limited number of studies assessing EMG outcomes following administration of cell-based therapies (Klimczak et al. 2020). In the report by Klimczak et al. (2020), on three DMD patients, authors confirmed an improvement in some of the EMG parameters at 6 months following local therapy administration. However, there are several differences when compared with our study, including systemic delivery of cell therapy, increased number of the tested EMG parameters and a longer, 12-month follow-up. Despite these differences, both studies confirm the value of using EMG for the assessment of clinical outcomes after the administration of cell-based therapy in the DMD patients’ population (Klimczak et al. 2020).

There are some limitations of the study which need to be acknowledged. The EMG assessments in this first-in-human study are based on three DMD patients. Including a higher number of DMD patients to the clinical studies is challenging, considering DMD is a rare disease, and the limited number of patients who would fit into the inclusion criteria of the study protocol. However, currently, there are additional patients enrolled in the DT-DEC01 study, and the collected EMG data will increase the statistical power of the study allowing for generalization of the results. Therefore, the ongoing studies will address these limitations. Furthermore, the EMG assessment was based on approximately ten MUP recordings from each tested muscle. Thus, for a more comprehensive interpretation of presented findings it would be beneficial to have longer recordings from a larger number of motor units. However, this task would be challenging in the pediatric patient’s population due to lack of attention and the fatigue over the extended time of EMG recordings.

It is important to note that despite these limitations, the presented findings suggest that treatment with the single dose of DT-DEC01 therapy is safe and effective up to 12 months after systemic–intraosseous administration. This is an important finding and provides promising evidence for the potential use of DT-DEC01 therapy as a treatment option for DMD patients. Furthermore, the improvement of the EMG parameters corresponding with the improvement of functional outcomes in response to DT-DEC01 therapy, confirms the use of electrophysiological assessments as a biomarker of changes occurring in the muscles of DMD patients after the therapy. This is a significant finding, as it provides researchers with a tool to assess efficacy of other therapies for DMD. Overall, while acknowledging the limitations of the study, the presented results are promising and provide important insights into the potential use of DT-DEC01 therapy as a treatment option for DMD patients. The ongoing studies with larger sample sizes and longer recordings will further confirm value of EMG as a biomarker of DT-DEC01 therapy efficacy in DMD patients.

## Conclusions

This study confirmed the long-term safety of DT-DEC01 therapy up to 22 months after systemic–intraosseous administration.

EMG assessment of selected muscles of the upper and lower extremities of both ambulatory and non-ambulatory DMD patients revealed a significant increase in the MUP duration and amplitudes maintained over the entire follow-up period up to 12 months after DT-DEC01 therapy administration.

The findings of this study demonstrate the significant benefits of DT-DEC01 therapy on skeletal muscle activity in DMD patients, confirmed by sustained improvement of the tested EMG parameters. Moreover, the increased MUP duration recorded in the selected muscles of the upper extremity correlated with the improved functional outcomes measured by the PUL 2.0 test and grip strength test in all three DMD patients, both ambulatory and non-ambulatory. Therefore, MUP duration was identified as a sensitive and reproducible biomarker of the restoration of muscle activity over a 12-month follow-up period. Hence, the use of EMG as a minimally invasive and objective evaluation represents a safe and effective method for assessing novel therapeutic approaches for DMD patients.

Finally, the results of this study confirming long-term improvement of skeletal muscle activity in ambulatory and non-ambulatory DMD patients are important, considering the progressive nature of the DMD and the lack of therapies that would either cure or halt the progression of the disease.

## Data Availability

All data generated or analyzed during this study are included in this published article.
